# Poster 1026: Randomized clinical trial to evaluate oral dializable leukocyte extracts as immunemodulator treatment in atopic dermatitis

**DOI:** 10.1186/1939-4551-7-S1-P16

**Published:** 2014-02-03

**Authors:** Alejandro Estrada García, Jorge Galicia Carreón, Erika Ramírez, Mirna Toledo, Sergio Estrada Parra, Mayra Pérez Tapia, María Del Carmen Jiménez Martínez

**Affiliations:** 1 National Polytechnic Institute, Mexico., Department of Immunology, National School of Biological Sciences, Mexico City, Mexico; 2 Faculty of Medicine, National Autonomous University of Mexico, Department of Immunology, Research Unit, “Conde de Valenciana” Foundation, Mexico, Mexico City, Mexico; 3 National Autonomous University of Mexico, Paediatrics Dermatology Department, Hospital Infantil de México Federico Gómez, Mexico, Mexico City, Mexico; 4 Universidad Nacional Autónoma de México, Faculty of Medicine, Mexico City, Mexico

## Background

To investigate efficacy and safety of Dialyzed Leukocyte Extracts (DLE) as adjuvant therapy for moderate atopic dermatitis (AD).

## Design

Double blind, placebo-controlled, randomized, clinical single-centre trial.

## Methods

Fifty-eight paediatric-patients with moderate AD were enrolled and randomized in two groups: 1) Conventional treatment (CT) + DLE: Cetirizine (0.25mg/kg), once daily/4 weeks, Chlorpheniramine (0.35 mg/Kg), daily divided in 3 doses/4 weeks; and topical Methylprednisolone 0.1%, twice daily/10 days, then once daily/10 days, and ending every 48h/10 days; plus oral DLE (2mg/5mL), daily/5 days, then every 72h to complete one month. 2) CT + placebo, same dosage and administration. Main outcome measures: Severity of skin lesions evaluated with SCORAD-Index, and immunophenotypical changes at day 14, and at end of treatment.

## Results

A significant clinical improvement was observed since day 14 with both, CT and CT + DLE therapy, no significant differences in the main clinical outcome measures were found among groups; however a diminished frequency of CD8+ CD103+ cells, and increased frequency of CLA + cells, was observed in CT + placebo-group.

## Conclusions

Adjuvant therapy with DLE was safe and well-tolerated. Despite both groups of patients significant improved after treatment, individuals treated with DLE preserved cell subsets related to skin immunological regulation, and avoided systemic expansion of cells related to skin inflammation.

